# A Pioneer of Cardiothoracic Surgery — the Brazilian Northeast Heart Transplant Program

**DOI:** 10.21470/1678-9741-2024-0128

**Published:** 2025-05-20

**Authors:** Ricardo de Carvalho Lima, José Teles de Mendonça, José Wanderley Neto, Mozart Augusto Soares de Escobar, José Glauco Lobo Filho, José Ricardo Lagreca de Sales Cabral

**Affiliations:** 1 Department of Thoracic Surgery, Universidade Federal de Pernambuco (UFPE), Recife, Pernambuco, Brazil; 2 Department of Thoracic Surgery, Universidade de Pernambuco (UPE), Recife, Pernambuco, Brazil; 3 Department of Cardiovascular Surgery, Universidade Federal de Sergipe (UFSE), Aracajú, Sergipe, Brazil; 4 Department of Surgery, Universidade Federal de Alagoas (UFAL), Maceió, Alagoas, Brazil; 5 Department of Surgery, Universidade Federal de Pernambuco (UFPE), Recife, Pernambuco, Brazil; 6 Department of Surgery, Universidade Federal do Ceará (UFCE), Fortaleza, Ceará, Brazil; 7 Department of Surgery, Universidade Federal do Rio Grande do Norte (UFRN), Natal, Rio Grande do Norte, Brazil

**Keywords:** Surgery, Pioneer, Heart Transplantation, Historical Article

## Abstract

This review highlights the pivotal milestones in the development of cardiac transplantation and related techniques. Beginning with Alexis Carrel's pioneering work on vascular anastomosis and organ preservation, the narrative progresses through groundbreaking achievements such as John Gibbon's invention of the heart-lung machine in 1953 and James Hardy's daring chimpanzee-to-human heart transplant in 1964. The story culminates in Christiaan Barnard’s historic human heart transplant in 1967 and Euryclides Zerbini's leadership in bringing this innovation to Brazil in 1968. Key advancements include the development of orthotopic heart transplantation techniques by Richard Lower and Norman Shumway and the resurgence of heart transplants following the introduction of cyclosporine in 1983, which revolutionized organ rejection management. The collaborative Programa Nordeste de Transplante Cardíaco, initiated in 1986, exemplifies regional innovation in overcoming logistical and financial barriers in Brazil. Recent progress, such as the first successful xenotransplantation using a genetically modified pig heart in 2022, underscores ongoing efforts to address donor shortages and improve transplant outcomes. This narrative is a testament to human ingenuity and perseverance in offering life-saving solutions to end-stage heart disease.

## INTRODUCTION

**Fig. 1:**
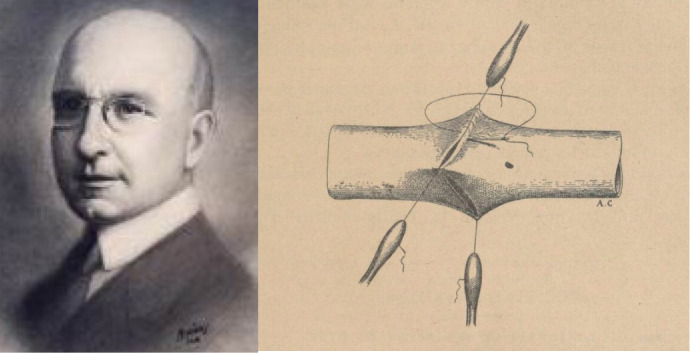
Alex Carrel received the Nobel Prize for creating the vascular suture technique.

**Fig. 2:**
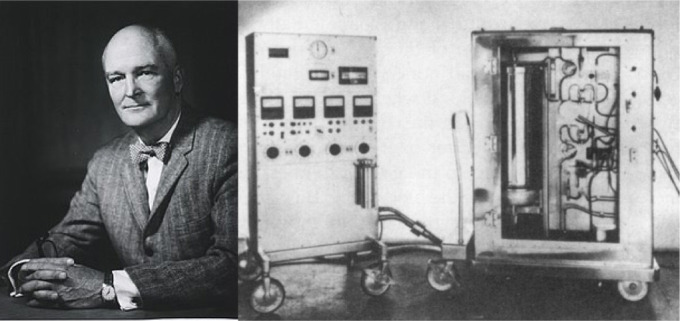
John Gibbon and his heart-lung machine used in 1953 to close atrial septal defect.

The possibility of carrying out the world’s first heart transplant in 1967 only became reality after two important previous contributions: the initial was in 1902, by Alex Carrel (Figure 1), introducing the technique of vascular suture into the universe of surgery^[^1^]^, and the other happened 51 years later (in 1953), when John Gibbon (Figure 2) developed the first artificial heart-lung machine, which temporarily allowed the diversion of blood, allowing to stop the heart and lungs during heart surgery, ensuring blood circulation and oxygenation throughout the body^[^2^]^.

## VASCULAR ANASTOMOSIS TECHNIQUE

Carrel’s research was mainly concerned with experimental surgery and transplantation of tissues and whole organs. As early as 1902, he published, in the Lyon Medical Journal^[^3^]^, a technique for the end-to-end anastomosis of blood vessels. Earlier, in 1908, he had devised methods for the transplantation of whole organs and two years later, in 1910, he demonstrated that organs could be kept for long periods in cold storage before they were used as transplants in surgery, and many experiments were performed on animal kidney transplants. Later on, in 1935, and in collaboration with Charles Lindbergh (the airman who was the first to flow across the Atlantic Ocean), he devised a machine for supplying a sterile oxygenation system to organs removed from the body. Lindbergh solved the mechanical problems involved, and Carrel published it in his book *“The Culture of Whole Organs”*^[^4^]^.

## EXTRACORPOREAL CIRCULATION

In 1953, Jonh H. Gibbon Jr, in Philadelphia, performs the first successful human heart surgery assisted by a heart-lung machine on an 18-year-old patient with an atrial septal defect and a significant left-to-right shunt. For 26 minutes, Gibbon kept his patient’s heart connected with the heart-lung machine using the patient’s arteries and veins. The blood circulated into a pump machine and oxygenator, getting back to the patient. The operation was the first in the world to use a heart-lung machine temporarily replacing the heart and lungs and their functions of the cardiopulmonary system^[^5^]^.

## THE BIRTH OF HEART TRANSPLANTATION

### First Heart Transplant in the World using Animal Heart (Xenotransplantation)

**Fig. 3:**
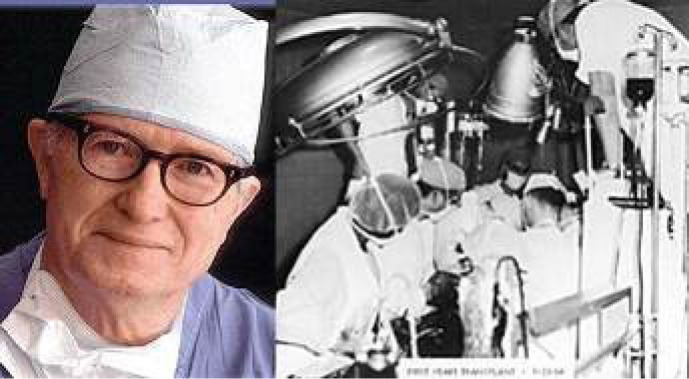
James Hardy, pioneer of the human heart transplant.

In the 1960s, at the University of Mississippi, James Hardy (Figure 3) performed heart transplants in a very large number of animals. Hardy and his team performed the first heart transplant into a human in an era that brain death was not yet recognized. In 1964, the patient — with lower extremity gangrene, hypertension, and a history of multiple myocardial infarction — was dying of cardiac failure, and a possible donor could not be brought together. The patient's hemodynamic situation deteriorated, he was taken to the operating room, and the heart of a chimpanzee was transplanted into him. The chimpanzee heart sustained a blood pressure of 90-100 mm/Hg for 90 minutes off cardiopulmonary bypass, but the patient died due a combination of an undersized heart and metabolic derangement. This courageous and challenging decision proved that it was feasible to perform a heart transplant in a human^[^6^]^.

### Orthotopic Heart Transplantation Technique

**Fig. 4:**
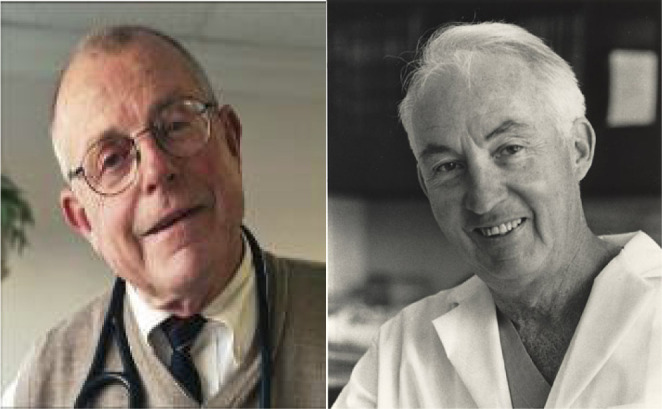
Richard Lower (left) and Norman Shumway (right) developed the technique that has been the gold standard for orthotopic heart transplantation until nowadays.

Richard R. Lower and Norman E. Shumway (Figure 4) were the pioneer cardiac surgeons responsible for the development of *orthotopic heart transplantation techniques*. These surgeons honed methods for heart transplantation in dogs, transplanting a canine heart from one dog to another. In 1959, they reported xenotransplantation developing a new technique that allowed a dog to live for eight days post-transplantation. In the next year, they published what has been called the *cardinal paper in orthotopic cardiac transplantation*, in which they combined surgical advances with improvements in recipient support and donor organ preservation. On January 6, 1968, Shumway undertook the third heart transplantation in the United States of America, and Lower, in May 1968, performed his own first human heart transplantation in human^[^7^,^8^]^.

### First Heart Transplant in Human

**Fig. 5:**
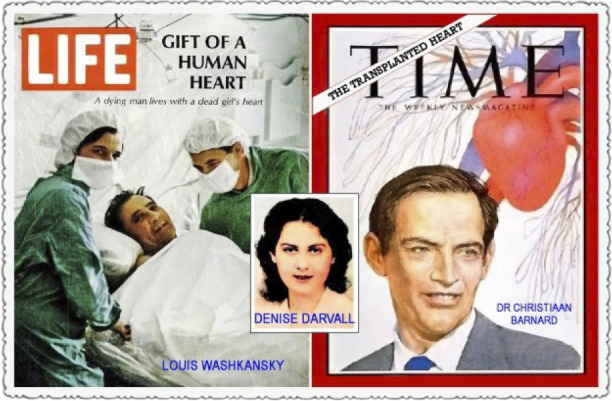
Christiaan Barnard and the first donor (center) and recipient of heart transplant (left) in December 3, 1967. Barnard said: Her heart was willing, but his body was weak.

The first human heart transplant had been carried out by Christiaan Barnard at Groote Schuur Hospital, in Cape Town/South Africa, on December 3, 1967 (Figure 5). Before this magnificent feat, Barnard went to Richmond, Virginia, and took a three-month sabbatical to gain experience in immunosuppressive therapy in patients with kidney transplants, which he did by participating in the transplant program headed by David Hume^[^9^]^. He gained more experience of experimental heart transplantation in the laboratory of Richard Lower who had trained with Norman Shumway. One of the contributing factors for Barnard accomplished this unprecedented feat was the difference in the definition of death at the time. In South Africa, doctors could already declare a patient dead when brain death was found, making the path to organ donation easier. On the other hand, in the United States of America, only the absence of a heartbeat made the diagnosis of death valid. Barnard had immense confidence and courage in undertaking this first operation in December 3rd, 1967^[^10^,^11^]^.

### Second Heart Transplant in the World

**Fig. 6:**
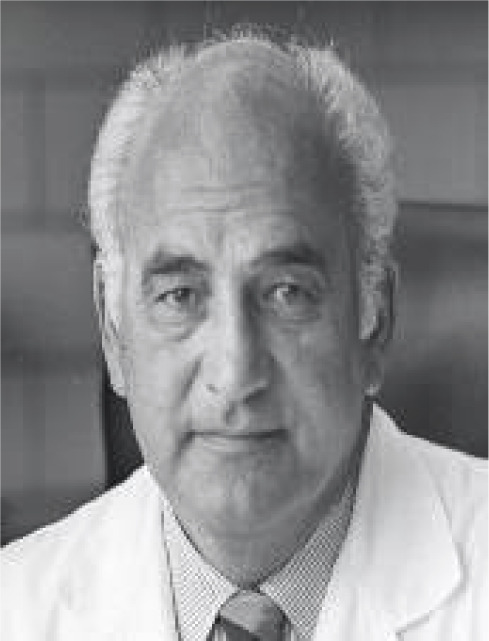
Adrian Kantrowitz performed the world's second human heart transplant in the world and the first in the United States of America.

Three days after Barnard’s first transplant, on December 6, 1967, Adrian Kantrowitz (Figure 6) and his surgical team at Maimonides Hospital in Brooklyn, New York/United States of America, performed the world's second heart transplant, after an aborted attempt in the previous year. Kantrowitz and his colleagues were given approval to do a heart transplantation using the heart of an anencephalic newborn donor in a 19-day-old recipient with severe congenital heart disease who survived for just six and a half hours after the operation. In the next month, Kantrowitz did another heart transplantation — the fifth in the world — this time in an adult, who also died within hours^[^12^]^.

### First Heart Transplant in Brazil and Latin America

**Fig. 7:**
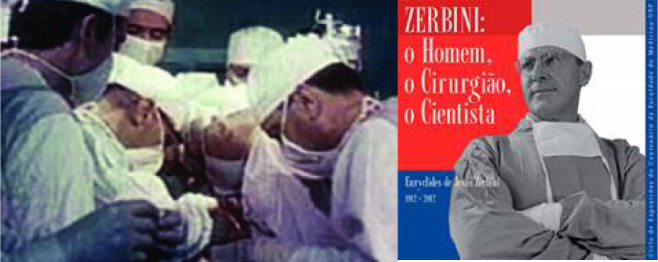
Zerbini and his surgical team (left) performing the first heart transplant in Brazil, 1968. The man, the surgeon, the scientist (right).

Five months after Barnard’s first heart transplant (May 26, 1968), Brazil joined the list of pioneering countries in heart transplantation in the world. At the Hospital das Clínicas of the Universidade de São Paulo, Brazil, the medical team led by Professor E. J. Zerbini (Figure 7) was responsible for the first heart transplant in Brazil and Latin America. The recipient was a patient with dilated cardiomyopathy, and the donor was a patient with severe cerebral trauma. The patient had an excellent recovery and died on the 28^th^ postoperative day due to rejection. The second patient transplanted by Zerbini, with ischemic heart failure, had survival a little more than one year in excellent clinical condition. The third patient operated on died on the 60^th^ day from infection^[^13^]^. Zerbini and his team were prepared to perform a heart transplant in 1967 and could have been the pioneer in the world performing the first heart transplant before Barnard, however, at that time, there was great difficulty in defining brain death among doctors, which made it impossible for this historic achievement to have a Brazilian paternity. As worldwide controversy raged over the ethics of heart transplantation, which after one year and 101 procedures still seemed of questionable benefit, this period of euphoria in several surgical cardiac centers around the world ended, and the transplant era was interrupted due to the lack of immunosuppressive drugs^[^13^,^14^]^.

## DISCOVERY OF CYCLOSPORINE AND THE SECOND TRANSPLANT ERA

In 1972, a compound derived from fungi called cyclosporine was able to suppress the rejection of transplanted organs without major damage to the recipient's immune system, allowing the evolution of this type of procedure throughout the world. It was by chance that in 1971, Professor Jean-François Borel, using samples of the fungus *Tolypocladium inflatum*, identified its immunosuppressive capacity on the immune system, responsible for organ rejection. Cyclosporine was introduced for clinical use in 1983, allowing solid organ transplantation with unexpected results, especially in heart transplantation. Its use in transplants has become routine, enabling the resumption of various transplants around the world^[^15^,^16^]^.

### Reintroduction of Heart Transplants in Brazil — The Cyclosporine Era

**Fig. 8:**
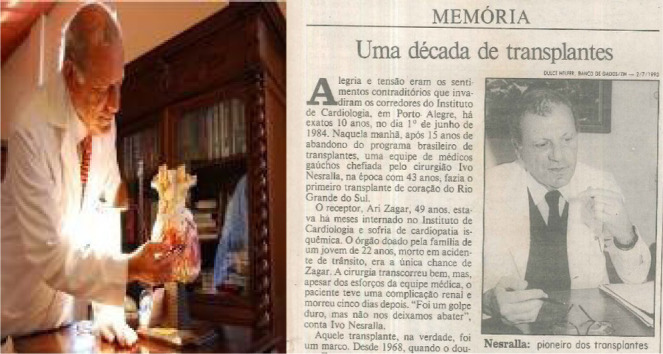
After 16 years of Zerbini achievement, Ivo Nesralla performed the second heart transplant in Brazil using cyclosporine.

In June 1, 1984, after 16 years of interruption of heart transplantation around the world, the team from the Instituto de Cardiologia do Rio Grande do Sul (Brazil), led by Ivo Abrahão Nesralla (Figure 8), resumed heart transplant at the cyclosporine era in Brazil. After the introduction of cyclosporine into the therapeutic arsenal against rejection, the heart transplant was reintroduced in different cardiac centers around the world^[^17^]^.

### First Heart Transplant in Northeast Brazil

**Fig. 9:**
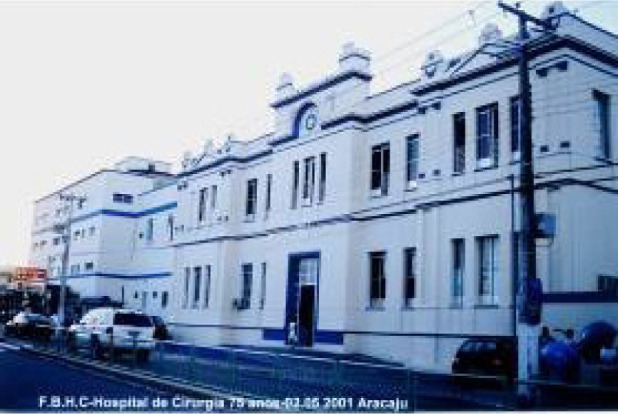
The Hospital de Cirurgia in Aracajú, Sergipe, where the first heart transplant of the Brazilian Northeast region was performed.

**Fig. 10:**
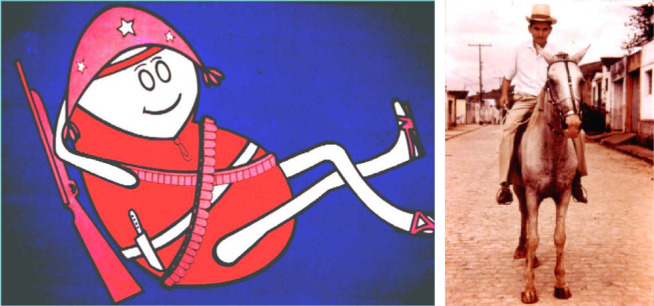
Brand of the Programa Nordeste de Transplante Cardíaco (or Northeast Heart Transplant Program) created by Teles and Wanderley in 1988 (left) and the oldest heart transplanted patient in Brazil (died in 2022, due to a cancer).

On June 19, 1986, José Teles de Mendonça together with José Wanderley performed the first heart transplant in the Northeast region of Brazil at the Hospital de Cirurgia, in Aracajú, Sergipe/Brazil (Figure 9). The patient was a 25-year-old male with diagnosis of Chagas disease. After achieving this historic regional feat, Teles and Wanderley designed a innovative heart transplant programmer denominated Programa Nordeste de Transplante Cardíaco (or Northeast Heart Transplant Program) (Figure 10) with intention of developing heart transplantation in the whole Brazilian Northeast region^[^18^]^.

### Programa Nordeste de Transplante Cardíaco

At that time, there were extreme difficulties in heart donation due to the lack of awareness among the population and the lack of understanding by the doctors of the concept of brain death followed by organ donation. It was a great difficulty to overcome in order to perform a heart transplant. At that period, the Sistema Nacional de Transplante (or National Transplant System), coordinated by the Ministry of Health, did not exist, and the Sistema Único de Saúde (or Unified Health System) did not finance the hospitals and professionals involved. The basic concept of the program was to integrate several regional centers with the aim of optimizing the number of heart transplants. The patient with diagnosis of terminal heart disease was placed on a single regional waiting list involving several states in the Northeast of Brazil.

The integrated centers located in different states started working together using a single pre-established protocol, widely studied and discussed among team members. Following the transplant protocol, the recipient was allowed to join the program and placed on the regional waiting list. The units involved at the program were: Hospital de Cirurgia (Sergipe), Santa Casa de Misericórdia (Alagoas), Real Hospital Português - UNITÓRAX (Pernambuco), Hospital Português (Bahia), Hospital Universitário Onofre Lopes (Rio Grande do Norte), and Hospital Antonio Prudente (Ceará). The patients were selected in their original center and registered on a general single waiting list composed of patients from all transplant center units. After this point, all centers start to search for an organ donor in their state. Once the donor was identified in another state, the recipient was transported to the state where the donor was located. The heart transplant was performed at the hospital where the donor was hospitalized. The lack of resources did not allow a capture team to be transferred to another state to collect the donor heart, and moving the recipient to the location where the donor was located was cheaper way, and expenses could be covered by members of the surgical team. This creative idea allowed the transplant to be carried out without mobilizing the donor, avoiding ischemia-like hemodynamic instability in the donor organ. After the transplant, the patient remained at the transplant donor center for approximately 30 days, and then returned to his/her original state hospital, starting the period of late follow-up at the origin transplant unit.

**Fig. 11:**
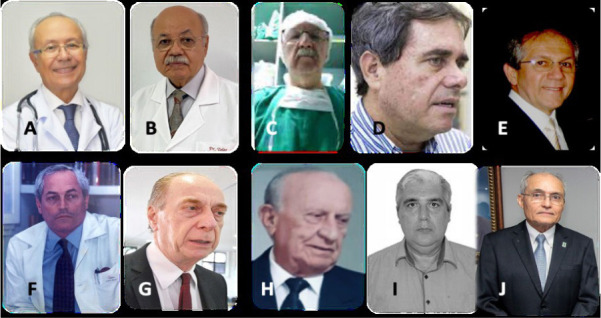
Coordinators of the Programa Nordeste de Transplante Cardíaco (or Northeast Heart Transplant Program) and their respective Brazilian city/state. A) José Wanderley (Maceió, Alagoas), B) José Teles (Aracajú, Sergipe), C) Luiz Daniel Torres (Maceió, Alagoas), D) Marcos Ramos (Aracajú, Sergipe), E) Ricardo Lima (Recife, Pernambuco), F) Mozart Escobar (Recife, Pernambuco), G) Ricardo Lagreca (Natal, Rio Grande do Norte), H) André Nunes (Natal, Rio Grande do Norte), I) Paulo Porciúncula (Salvador, Bahia), and J) José Glauco (Fortaleza, Ceará).

**Fig. 12:**
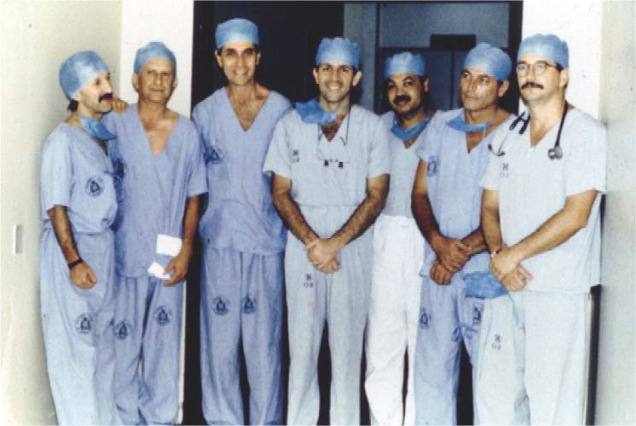
The surgical team of the Programa Nordeste de Transplante Cardíaco (or Northeast Heart Transplant Program) who performed the first heart transplant in Recife on October 18, 1991. From left to right the surgeons: Daniel Torres (Alagoas), André Nunes (Rio Grande do Norte), Ricardo Lagreca (Rio Grande do Norte), Ricardo Lima (Pernambuco), José Teles (Sergipe), Mozart Escobar (Pernambuco), and Roberto Alecrim (Pernambuco).

The Programa Nordeste de Transplante Cardíaco was a pioneer in heart transplantation in several states of Brazil: Sergipe, Alagoas, Pernambuco, Bahia, and Ceará. In a harmonious way, this cooperation between the various transplant centers not only allowed the development of transplants, increasing the number of transplants and increasing the team experience in other areas of cardiac surgery, but also managed to overcome financial difficulties to capture hearts at long distance. The surgical team (Figures 11 and 12) paid with its own resources the recipient transportation to the donor location and also the patient’s return to the hospital of origin^[^18^]^.

### Other Programs in Northeast Brazil

After 38 years of the historic milestone in carrying out the first heart transplant in the Northeast Brazil and the creation of the unique Programa Nordeste de Transplante Cardíaco by Teles and Wanderley, other programs were developed also, including those by the Instituto do Coração de Pernambuco/Real Hospital Português, coordinated by Carlos Moraes^[^19^]^; Hospital Mesejana, coordinated by Juan Alberto Cosquillo Mejía^[^20^]^; Instituto de Medicina Integral Professor Fernando Figueira, coordinated by Fernando Figueira Filho; and, more recently, PROCAPE – Hospital do Coração/UPE, coordinated by Frederico Brownie.

## A HUGE STEP FOR HUMANITY

Bartley P. Griffith in 2022 performed a successful genetically modified pig heart transplant on a 57-year-old man with terminal heart disease. The historic surgery was conducted at the University of Maryland Medical Center. This organ transplantation demonstrated for the first time that a genetically modified animal heart (xenotransplant) can function like a human heart without immediate rejection by the body. Griffith said this innovative surgery brings one step closer to solving the world's organ shortage crisis. About 110,000 Americans are currently waiting for an organ transplant, and more than 6,000 patients/year die on the waiting list. Xenotransplantation could save thousands of lives in the future, although it still presents a set of risks, including immediate and fatal rejection^[^21^]^.

## COMMENTS

The saga of cardiac surgeons and heart transplantation in the world is a captivating tale of medical breakthroughs and life-saving procedures. It is a story that spans decades and shows the relentless pursuit of knowledge and innovation in the field of cardiac surgery. The journey of heart transplantation began with the pioneering work of surgeons like Alex Carrel, John Gibbon, James Hardy, Richard Lower, and Norman Shumway. But Dr. Christiaan Barnard performed the world's first successful heart transplant in 1967. Five months after the Barnard’s transplant, Zerbini, in 1968, performed the South America’s first successful heart transplant in Brazil. The first heart transplant performed in Northeast Brazil was performed by Teles and Wanderley, in 1986.

**Fig. 13:**
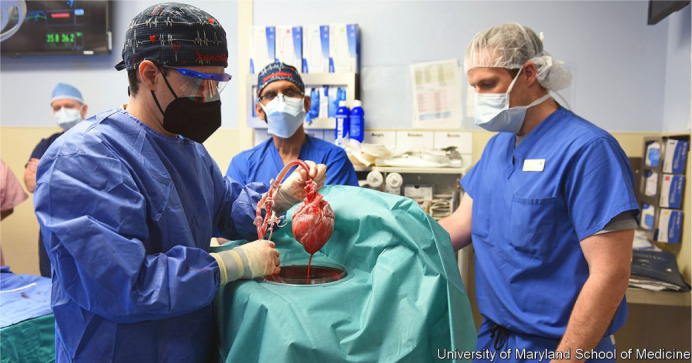
Dr. Griffith during the first successful genetically modified pig heart transplant in 2022, United States of America

This groundbreaking achievement opened new possibilities for patients suffering from end-stage heart disease, offering them a chance of a longer and healthier life. However, the early days of heart transplantation were fraught with challenges. The scarcity of suitable donor hearts, the risk of organ rejection, and the need for lifelong immunosuppressive medications posed significant hurdles for surgeons. Despite these obstacles, cardiac surgeons persevered, refining their techniques and improving patient outcomes. Over time, advancements in surgical techniques, immunosuppressive therapies, and organ preservation methods have greatly enhanced the success rates of heart transplantation. Surgeons have become adept at performing complex procedures, ensuring the safe removal of the donor heart, graft preservation, and its successful implantation in the recipient's chest. The saga of cardiac surgeons in heart transplantation also highlights the importance of collaboration and teamwork. Surgeons work closely with a multidisciplinary team of healthcare professionals, including cardiologists, anesthesiologists, nurses, immunologists, infectious disease specialists, technicians, and transplant coordinators to ensure the best possible outcomes for their patients. Furthermore, the saga extends beyond the operating room. Cardiac surgeons are involved in ongoing research and clinical trials, seeking to improve transplant techniques, develop new immunosuppressive therapies, and explore alternative options such as xenotransplantation (transplanting organs from animals). In December 2022, the first xenotransplantation in the world was carried out with an immediate success without immediate rejection, and this innovative surgery brings one step closer to solving the world's organ shortage crisis (Figure 13). Despite the remarkable progress made in heart transplantation, challenges remain. The shortage of donor organs continues to be a significant issue, leading to long waiting lists and the need for innovative solutions such as organ preservation technologies and artificial hearts.

And in 1986, two daring young surgeons (Teles and Wanderley), working in one of the poorest regions of Brazil, devised an original heart transplant program with the aim of increasing the numbers of transplants, offering this sophisticated form of treatment for poor people and developing cardiac surgery in the entire region. Today, several isolated centers perform heart transplants in the North-Northeast Brazil with hundreds of heart patients transplanted.

Since James Hardy performed the first human transplant on a dying patient by implanting a chimpanzee heart in a man and the concept of brain death did not exist, 65 years have been passed for this feat to be accomplished again. Many authors and collaborators around the world that began by overcoming cultural difficulties, religious paradigms, graft rejection, and graft protection have been overcome with the huge performance of the first human xenotransplant in 2022. This extraordinary accomplishment opened enormous perspectives in the future for the treatment of terminal cardiomyopathy, offering a possible solution for donor shortage and solving the difficulties that still exist in current days related to the donor supply and the major number of recipients.

## CONCLUSION

The saga of cardiac surgeons in heart transplantation is a testament to human ingenuity, perseverance, and compassion. Through their tireless efforts, these surgeons have transformed the lives of countless patients, offering them a second chance at life and inspiring hope for a brighter future in the field of cardiac surgery like genetically modified xenotransplantation.

**Table:** 

Authors’ Roles & Responsibilities	
RCL	Substantial contributions to the conception or design of the work; or the acquisition, analysis, or interpretation of data for the work; drafting the work or revising it critically for important intellectual content; agreement to be accountable for all aspects of the work in ensuring that questions related to the accuracy or integrity of any part of the work are appropriately investigated and resolved; final approval of the version to be published
JTM	Substantial contributions to the conception or design of the work; or the acquisition, analysis, or interpretation of data for the work; drafting the work or revising it critically for important intellectual content; agreement to be accountable for all aspects of the work in ensuring that questions related to the accuracy or integrity of any part of the work are appropriately investigated and resolved; final approval of the version to be published
JWN	Substantial contributions to the conception or design of the work; or the acquisition, analysis, or interpretation of data for the work; drafting the work or revising it critically for important intellectual content; agreement to be accountable for all aspects of the work in ensuring that questions related to the accuracy or integrity of any part of the work are appropriately investigated and resolved; final approval of the version to be published
MASE	Substantial contributions to the conception or design of the work; or the acquisition, analysis, or interpretation of data for the work; drafting the work or revising it critically for important intellectual content; agreement to be accountable for all aspects of the work in ensuring that questions related to the accuracy or integrity of any part of the work are appropriately investigated and resolved; final approval of the version to be published
JGLF	Substantial contributions to the conception or design of the work; or the acquisition, analysis, or interpretation of data for the work; drafting the work or revising it critically for important intellectual content; agreement to be accountable for all aspects of the work in ensuring that questions related to the accuracy or integrity of any part of the work are appropriately investigated and resolved; final approval of the version to be published
JRLSC	Substantial contributions to the conception or design of the work; or the acquisition, analysis, or interpretation of data for the work; drafting the work or revising it critically for important intellectual content; agreement to be accountable for all aspects of the work in ensuring that questions related to the accuracy or integrity of any part of the work are appropriately investigated and resolved; final approval of the version to be publishe

## References

[1] Carrel A (2023). Facts. NobelPrize.org. Nobel Prize Outreach AB.

[2] Hill JD (1982). John H. Gibbon, Jr. Part I. The development of the first
successful heart-lung machine. Ann Thorac Surg..

[3] Carrel A, Morel B (1902). Anastomose bout a bout de la jugulaire et de la carotide
primitive. Lyon Med..

[4] Carrel A (1937). The culture of whole organs : I. techinique of the culture of the
thyroid gland. J Exp Med..

[5] Gibbon JH (1978). The development of the heart-lung apparatus. Am J Surg..

[6] The University of Mississippi Medical Center (c2023). Dr James D. Hardy [Internet].

[7] Oransky I (2006). Norman Shumway. Lancet.

[8] Pincock S (2008). Richard Rowland Lower. Lancet.

[9] Klintmalm GB (2004). The history of organ transplantation in the Baylor health care
system. Proc (Bayl Univ Med Cent).

[10] Barnard CN (1967). The operation. A human cardiac transplant: an interim report of a
successful operation performed at Groote Schuur Hospital, Cape
Town. S Afr Med J..

[11] Cooper DKC (2018). Christiaan Barnard-The surgeon who dared: the story of the first
human-to-human heart transplant. Glob Cardiol Sci Pract..

[12] Harding A (2009). Adrian Kantrowitz. Lancet.

[13] Stolf NA, Braile DM (2012). Euryclides de Jesus Zerbini: a biography. Rev Bras Cir Cardiovasc..

[14] Lima R, Lucchese FA, Braile DM, Salerno TA (2012). A tribute to Euryclides de Jesus Zerbini, MD. Rev Bras Cir Cardiovasc..

[15] Javier MFDM, Javier Delmo EM, Hetzer R (2021). Evolution of heart transplantation since Barnard's
first. Cardiovasc Diagn Ther..

[16] Heusler K, Pletscher A (2001). The controversial early history of cyclosporin. Swiss Med Wkly.

[17] Rodrigues da Silva P (2008). Transplante cardíaco e cardiopulmonar: 100 anos de
história e 40 de existência. Rev Bras Cir Cardiovasc..

[18] Lima R, Escobar M, Alecrim R, Alves I, Lins T, Arraes N (1992). Programa Nordeste para transplante cardiaco “NETx:
experiência atual. Braz J Cardiovasc Surg..

[19] Moraes Neto F, Tenório D, Gomes CA, Tenório E, Hazin S, Magalhães M (2001). Transplante cardíaco: a experiência do instituto do
coração de Pernambuco com 35 casos. Braz J Cardiovasc Surg..

[20] Vieira JL, Sobral MGV, Macedo FY, Florêncio RS, Almeida GPL, Vasconcelos GG (2022). Long-term survival following heart transplantation for Chagas
versus non-Chagas cardiomyopathy: a single-center experience in Northeastern
Brazil over 2 decades. Transplant Direct..

[21] Griffith BP, Goerlich CE, Singh AK, Rothblatt M, Lau CL, Shah A (2022). Genetically modified porcine-to-human cardiac
xenotransplantation. N Engl J Med..

